# Exploring the Potential of Postbiotics for Food Safety and Human Health Improvement

**DOI:** 10.1155/2024/1868161

**Published:** 2024-08-06

**Authors:** Folayemi Janet Isaac-Bamgboye, Chiamaka Linda Mgbechidinma, Helen Onyeaka, Ireoluwa Toluwalase Isaac-Bamgboye, Deborah C. Chukwugozie

**Affiliations:** ^1^ Department of Chemical Engineering University of Birmingham, Birmingham, UK; ^2^ Department of Food Science and Technology Federal University of Technology, Akure, Ondo State, Nigeria; ^3^ Centre for Cell and Development Biology and State Key Laboratory of Agrobiotechnology School of Life Sciences The Chinese University of Hong Kong, Shatin, New Territories, Hong Kong, China; ^4^ Ocean College Zhejiang University, Zhoushan 316021, Zhejiang, China; ^5^ Department of Microbiology University of Ibadan, Ibadan, Oyo State 200243, Nigeria; ^6^ Department of Food Technology University of Ibadan, Ibadan, Oyo State, Nigeria; ^7^ Department of Microbiology Federal University Otuoke, Bayelsa State, Otuoke, Nigeria

## Abstract

Food safety is a global concern, with millions suffering from foodborne diseases annually. The World Health Organization (WHO) reports significant morbidity and mortality associated with contaminated food consumption, and this emphasizes the critical need for comprehensive food safety measures. Recent attention has turned to postbiotics, metabolic byproducts of probiotics, as potential agents for enhancing food safety. Postbiotics, including organic acids, enzymes, and bacteriocins, exhibit antimicrobial and antioxidant properties that do not require live organisms, and this offers advantages over probiotics. This literature review critically examines the role of postbiotics in gut microbiome modulation and applications in the food industry. Through an extensive review of existing literature, this study evaluates the impact of postbiotics on gut microbiome composition and their potential as functional food ingredients. Research indicates that postbiotics are effective in inhibiting food pathogens such as *Staphylococcus aureus, Salmonella enterica*, and *Escherichia coli,* as well as their ability to prevent oxidative stress-related diseases, and they also show promise as alternatives to conventional food preservatives that can extend food shelf life by inhibiting harmful bacterial growth. Their application in functional foods contributes to improved gut health and reduced risk of foodborne illnesses. Findings suggest that postbiotics hold promise for improving health and preservation by inhibiting pathogenic bacteria growth and modulating immune responses.

## 1. Introduction

Food safety is a critical concern globally, with millions of cases of foodborne illnesses reported each year. According to the World Health Organization (WHO), an estimated 600 million people (which is about 1 in 10 people in the world) fall ill annually after consuming contaminated food with 420,000 deaths worldwide, resulting in the loss of 33 million healthy life years (DALYs) [1]. These statistics highlight the urgency of ensuring food safety at all stages of production, processing, distribution, and consumption.

In recent years, there has been increasing interest in novel approaches to enhance food safety, one of which involves exploring the potential of postbiotic compounds. Postbiotics, the metabolic byproducts of probiotics in the gut, have emerged as promising agents for improving food safety. These groups of bioactive compounds are produced by probiotic microorganisms during fermentation [2]. They include organic acids, enzymes, bacteriocins, short-chain fatty acids, and peptides that have been shown to possess a wide range of benefits, including the inhibition of pathogens, reduction of inflammation, and immune system modulation [3]. Contrary to probiotics which require a live organism to exhibit their health benefits, postbiotics are nonviable and can be consumed in supplements or foods. In addition, postbiotics have antimicrobial and antioxidant properties, which can help improve food's shelf life and safety [4].

The antimicrobial properties of postbiotics are one of their significant benefits. According to a recent study by Noori *et al.* (2022, In Press), postbiotics can effectively inhibit the growth of foodborne pathogens such as *Staphylococcus aureus, Salmonella enterica*, and *Bacillus cereus.* İncili et al. [5] also investigated the antimicrobial activity of lactic acid bacteria (LAB) postbiotics against *Escherichia coli,* which is the most important pathogenic bacteria in foods, and found that a combination of chitosan and LAB postbiotics could effectively inhibit the growth of *E. coli*. In another study by Peng et al. [6], the postbiotic of *Lactobacillus casei* effectively controlled pathogens and reduced *E. coli* by 99%. This makes them an alternative to conventional antibiotics, often used in developing antibiotic-resistant strains of bacteria, and also as an alternative to conventional food preservatives. Postbiotics also have antioxidative properties that can prevent oxidative stress, which can damage cells leading to the development of chronic diseases such as diabetes, cancer, and cardiovascular diseases. By doing this, postbiotics play a crucial role in preventing chronic diseases hence maintaining good health [7]. In addition, postbiotics have food safety properties, making them an alternative to conventional food preservatives. They help in preventing harmful bacteria growth, which in turn reduces the spoilage of food and extends the shelf life of food products. This plays a crucial role in food safety [7], promoting good health.

As previously documented, dairy propionibacteria, the dominant starter cultures particularly in Swiss (hard) cheese production, demonstrate probiotic properties that stimulate the immune system and reduce blood cholesterol levels, among other *in vivo* functional favourable effects such as exhibiting a broad spectrum of antimicrobial activities and effectively inhibiting the growth of Gram-positive and some Gram-negative bacteria, certain yeasts, and molds [8]. Probiotic yoghurt (*L. acidophilus* and *B. lactis*) compared to regular yoghurt (*S. thermophilus* and *L. bulgaricus*) improves glucose control in women with gestational diabetes mellitus during pregnancy, a condition that can lead to complications for both the newborn and the overall outcome of the pregnancy [9]. This regimen may contribute to a reduction in the occurrence of macrosomia (large birth weight) in infants. Nevertheless, the use of probiotics has been associated with the spread of antibiotic-resistant genes within the existing microbial community. Recent research has yielded conflicting findings, suggesting that the viability of probiotic bacteria may not be necessary for them to exert positive effects on health [10]. The prevailing understanding is that probiotic foods primarily consist of *Lactobacillus* and *Bifidobacterium* species, which have only been sporadically linked to clinical diseases in humans. Although there may be potential risks associated with certain populations, probiotic products are generally regarded as safe food items in the market. Despite the potential dangers associated with the presence of bacteria in food, beneficial bacteria such as LAB and Bifidobacteria can outperform their pathogenic counterparts and contribute to the production of advantageous bioactive compounds [10]. Therefore, adopting strategies that could potentially alleviate the risks associated with probiotic consumption could prove to be advantageous.

Postbiotics are utilized in the food industry to develop functional foods; these are foods that go beyond basic nutrition to provide health benefits. In addition, postbiotics can improve the nutritional value of these foods and provide added health benefits [11]. Fermented foods containing postbiotics can reduce the risk of certain foodborne illnesses by improving gut health [12]. The study of postbiotics has been evolving rapidly and holds great promise for food safety and human health improvement. This study discusses postbiotics' production, sources and classes, properties, and association with the gut microbiome. The study also highlights food safety dynamics and postbiotics and considers the future perspective of postbiotics.

## 2. Postbiotics Production and Characterization Strategies

### 2.1. *In Vitro* Production Methods

Gut microbiota growth is largely dependent on host nutritional constituents, although capable of producing small molecular weight metabolites during their lifecycle to regulate growth, stress response, development, reproduction, and symbiotic association with other beneficial microbes [13]. Postbiotics are either secreted by live bacteria or released after bacterial lysis into the host environment, hence modifying the host cellular processes and metabolic pathways in a physiologically aided manner.

The type and quantity of postbiotic products depend largely on the bacterial strain, the culture medium, and the postgrowth processing of the bacteria. Postbiotics in food are not processed after growth and only have soluble factors, such as secreted products or metabolic waste, from the bacterial growth medium [14]. However, in some studies, bacterial cells are broken down after growth by cell disruption methods, such as heat, enzymes, chemicals, sonication, high pressure, solvent extraction, or a mix of these [15]. These methods release more intracellular metabolites and cell wall components into the postbiotic mix and give new properties to the resulting postbiotics. *In vivo* postbiotics and production strategies have been proven through several studies employing *in vitro* bioengineering procedures, mainly recombinant technologies, to boost the bioactive metabolite secretion [16].

The methods commonly employed to obtain postbiotics include cell disruption techniques, such as heat, and enzymatic treatments, solvent extraction, as well as sonication [13–15]. Each of the cell disruption techniques is used to produce postbiotics by breaking the cell membrane and releasing the bioactive compounds from the bacterial cells. However, the methods differ in the way they apply physical, chemical, or biological forces to the cells. Heat treatment involves exposing the bacterial cells to high temperatures, usually above 70°C, for a certain period. This causes the cell membrane to rupture and release the postbiotics [13]. Enzymatic treatments involve treating the bacterial cells with enzymes, such as lysozyme, protease, or cellulase, that digest the cell wall and membrane. This allows the postbiotics to be extracted more efficiently and selectively. In solvent extraction, the bacterial cells are dissolved in organic solvents, such as ethanol, methanol, or acetone, that dissolve the cell membrane and extract the postbiotics. In addition, sonication involves subjecting the bacterial cells to high-frequency sound waves that create cavitation bubbles which implode and disrupt the cell membrane. Sonication is a powerful and versatile method, but it may also generate heat and oxidative stress, which can damage the postbiotics. The choice of the cell disruption technique depends on several factors, such as the type of bacteria, the type of postbiotics, the yield, the cost, the stability, and the safety of the process [17]. To obtain a postbiotic fraction of *L*. *casei* with anticancer activity, Tiptiri-Kourpeti et al. [18] subjected *Lactobacillus casei* ATCC 393 to heat treatment at 100°C for 40 min, followed by sonication at 50 W for 10 min and centrifugation at 13,000 ×g for 40 min.

Industrial fermentation-based approaches are also popular for making postbiotics with potential health benefits. Postbiotics are mainly produced by bacteria and fungi from the genera of *Lactobacillus*, *Streptococcus*, *Bifidobacterium*, *Eubacterium*, *Saccharomyces*, and *Faecalibacterium*. Thorakkattu et al. [19] carried out a study that showed that postbiotics can be produced effectively by fermenting probiotic bacteria such as *Bifidobacterium* and *Lactobacillus* strains. These microbes generate a wide range of metabolites during fermentation, such as organic acids, peptides, and exopolysaccharides. These postbiotics have shown anti-inflammatory, antioxidant, and immunomodulatory effects [20]. Industrial fermentation enables the controlled production of these bioactive compounds, making it a feasible and eco-friendly method for making functional ingredients for various health-enhancing applications.

Table 1 presents a comparative analysis of different methods used for the preparation of postbiotics, highlighting their advantages and disadvantages. Fermentation is a scalable method that enhances the bioactivity of postbiotics, although it can be time-consuming and yield variable results [19]. Heat treatment is effective for ensuring sterility and is a quick process, but it can degrade sensitive compounds [18]. Ultrasonication is noted for its efficiency in cell disruption and speed, though it may affect the structural integrity of bioactive compounds [17].

In addition to the aforementioned extraction techniques, recent studies have reported the incorporation of centrifugation, dialysis, freeze-dried, column purification, and greener methods for higher yield recovery [15, 21]. A study by Jurášková et al. [22] described the extraction of exopolysaccharides (EPSs) from culture media/food matrices. Their approach involved several steps which include centrifugation to recover the EPS-containing supernatant, acid addition to remove high protein content, precipitation with cold ethanol, followed by dialysis/ultrafiltration, and finally freeze-drying to obtain a pure EPS solid.

Shafipour Yordshahi et al. [23] produced a postbiotic powder from *Lactobacillus plantarum* ATCC 14917 by lyophilization/freeze-drying, and they used it to impregnate bacterial cellulose to make a nanopaper that can prevent microbial growth on ground meat. According to a study by Tong et al. [24], postbiotics with potential health advantages for the gut were created through optimised solid-state fermentation. Postbiotics at a concentration of 25 mg/mL demonstrated substantial antioxidant activity against ABTS, DPPH, and OH radicals as well as notable broad-spectrum antibacterial activities against *Salmonella, Escherichia coli*, and *Staphylococcus aureus* in these ideal conditions. Furthermore, when it came to lowering nitric oxide (NO) release in RAW 264.7 macrophage cells in response to LPS-induced inflammation, the optimised postbiotics demonstrated strong anti-inflammatory properties. Moreover, the postbiotics markedly enhanced the capacity of intestinal epithelial wound repair following mechanical damage, such as scratches on IPEC-J2 cells (*p* < 0.05). Consequently, the results of Tong et al. [24] indicated that the novel postbiotics might be employed as prospective functional food products to enhance physical well-being.

### 2.2. Characterization Strategies for Postbiotics

Different analytical approaches exist for postbiotic identification, and their usage depends on the analytical goals and the type of characterization (qualitative and/or quantitative) required [19, 25, 26]. Commonly reported in research articles are spectroscopy methods in metabolomics, given the biological complexity of postbiotics having different polymerization degrees and glycosidic bonds. Gas chromatography (GC) is most frequently used for the quantitative and qualitative analysis of free fatty acids, volatile compounds (e.g., diacetyl, acetoin, dimethyl sulfone, and 2-butanone), and organic acids in postbiotics. In the design of synergistic delivery microcapsules for treating colitis mice, Yang et al. [27] utilized the GC-MS approach (GC-MS with an Agilent HP-INNOWax platform 7700A) to determine the overall richness and abundance of SCFA from *Faecalibacterium* and *Roseburia.* GC equipped with a flame ionization detector (GC-FID) can also be used to determine postbiotic SCFA. Liquid chromatography (LC), such as high-performance liquid chromatography (HPLC), having high potency, purity, efficiency, resolution, sensitivity, accuracy, and lesser solvent demand, is widely used for postbiotics qualitative and quantitative investigations. Toushik et al. [28] employed an Agilent HPLC 1260 Infinity equipped with a photodiode array detector to measure the organic acids in the LAB B67 postbiotic from *Lactobacillus curvatus* B67 that exhibits broad pH (1–6) and temperature (40–121°C) stability. Besides the performance features, HPLC is preferred as it allows for concomitant analysis of different organic acids (acetic, malic, lactic, acetic, and other acids) [13, 26] and possible coupling with other sophisticated equipment such as ultraviolet detector, ultraviolet-diode array detector (UV/DAD) refractive index (RI), mass spectroscopy, and pulsed electrochemical detection. Fourier transform infrared (FTIR) spectroscopy is recommended for qualitatively classifying the organic and inorganic components present in postbiotic metabolites. Jivkova et al. [29] described using FT-ICR-MS, NMR, and FTIR to determine the structural information of a synthesized novel exopolysaccharide (EPS) from *Ramlibacter tataouinensis* revealing the presence of saccharide and amino acids.

Other methods for postbiotic analysis include matrix-assisted laser desorption/ionization time-of-flight (MALDI-TOF) for protein identification, electrospray ionization mass spectrometry (ESI-MS) for metabolite molecular mass determination, two-dimensional gel electrophoresis 2D-PAGE for molecular weight determination, thin-layer chromatography (TLC) for qualitative testing, and spectrophotometric-based analysis for hydrogen peroxide concentration and total protein content measurement using colorimetric assays such as the Bradford method [15].

Gurunathan et al. [30] used the headspace solid-phase microextraction GC-MS method to identify the volatile compounds of postbiotics from *Lactobacillus casei*, and they detected sixty-two compounds. Likewise, GC was applied to measure the short-chain fatty acid levels of postbiotics from four different bacterial strains. HPLC is also a common analytical tool for the quantitative and qualitative assessment of postbiotics [31]. Ultra-performance liquid chromatography has superior performance in separating and identifying postbiotics, due to its high efficiency, resolution, sensitivity, and accuracy, as well as its low solvent consumption. Sharma et al. [32] and Wang et al. [33] employed TLC and verified the existence of various compounds in postbiotics. Moreover, colorimetric methods have been explored to determine the metabolite amounts in the postbiotics of LAB.

While these techniques are valuable for detecting, identifying, and quantifying postbiotics, further research is needed to improve extraction protocols, analytical tools, and optimization of culture conditions and media. This will enable the discovery and characterization of novel postbiotics and enhance our understanding of their mechanisms of action and modulation of signalling pathways.

#### 2.2.1. Applications in Food Safety

Food safety is threatened by numerous elements, including biological, chemical, and physical risks. Biological dangers are quite important in this aspect. Among these, bacteria are crucial in the deterioration of food and the development of foodborne illnesses. In addition, because probiotics and postbiotics have strong antibacterial qualities, a novel strategy centred on preventing the proliferation of pathogenic bacteria and their mediated corruption has been employed recently. According to the results of current studies, postbiotics may be suitable substitute components for probiotic cells and may be used as innovative antibacterial agents [34]. The *in vitro* production of postbiotics in food safety applications is a technique that aims to prevent the growth of potential foodborne pathogens and enhance the quality and shelf life of food products. Fermentation of dairy products, such as yoghurt, cheese, and kefir, with probiotic bacteria produces postbiotics, such as organic acids, bacteriocins, and exopolysaccharides, that can inhibit the growth of spoilage and pathogenic microbes, such as *Listeria, Salmonella*, and *Escherichia coli*.

Postbiotics primarily inhibit the growth of pathogenic microorganisms by forming pores in cell membranes, acidifying the cytoplasm of the cell blocking the production and regulation of energy, and altering the morphology and function of sensitive components such as proteins and peptides by oxidising bacterial cells and producing acidity in the bacterial cell membrane. Because of their special qualities, postbiotics are currently supported by scientific research as useful instruments in the food industry to prevent microbial deterioration and create functional foods [34].

Up until now, the majority of reports have concentrated on the usage of EPS and bacteriocins, two postbiotic metabolites, in food. Fish, meat, and related items are particularly vulnerable to bacterial infection, which can reduce their nutritional value, result in undesirable organoleptic changes, and endanger the health of consumers. Depending on the type of meat and the postbiotic's composition, postbiotics can be sprayed or coated directly onto meat and meat products to activate their antibacterial properties [35].

Recent research has also found that postbiotics can help eliminate harmful chemicals such as bisphenol A, pesticides, and mycotoxins. Bisphenol A is commonly found in foods such as fish, vegetables, meat, cheese, and wine. Certain types of bisphenol A, such as histamine, tyramine, putrescine, and cadaverine, can cause various health problems in humans [36]. GarcíaRuiz et al. [37] studied how certain bacteria found in wine, along with postbiotics, can break down bisphenol A in both laboratory settings and wine. They discovered that most of the bacteria they tested, primarily belonging to lactic acid bacteria, could break down at least two of the three types of bisphenol A they examined (HIS, TY, and PU) at the same time. In addition, they found that postbiotics from bacteria with a high bisphenol A-degrading ability were most effective at a pH of 4.7.

### 2.3. Common Classification of Postbiotics and Well-Recognized Sources

Postbiotics can be classified based on several factors including their elementary composition. This classification differentiates postbiotics into groups, i.e., lipids (e.g., butyrate, propionate, and dimethyl acetyl-derived plasmalogen), proteins (e.g., lactocepin and p40 molecule), carbohydrates (e.g., galactose-rich polysaccharides and teichoic acids), vitamins/cofactors (e.g., B-group vitamins), organic acids (e.g., propionic and 3-phenyllactic acid), and complex molecules such as peptidoglycan-derived muropeptides and lipoteichoic acids [16]. Following the elementary composition classification, Toushik et al. [28] grouped the postbiotics derived from *Lactobacillus curvatus* B67 into three major categories showing the predominant metabolic constitute as organic acids, lactic and acetic acids; amino acids, L-valine, L-alanine, lysine, tyramine, glycine, and L-threonine. Among lipids, SCFAs are attractive candidates for preventing and treating gastrointestinal diseases, including colitis, metabolic disorders, and cancer, due to their gut barrier governing potentials by regulating the gut microbiota and decreasing inflammation via histone deacetylases inhibition and histone acetyltransferases activation in colonocytes [27]. This suggests SCFA involvement in acetylation homeostasis within the nucleus, an essential property for neuronal vitality usually explored to ameliorate neurodegenerative diseases. Another classification basis is the physiochemical features, i.e., chemical structures, safety dose parameters, and longer shelf life [26].

**Figure 1:**
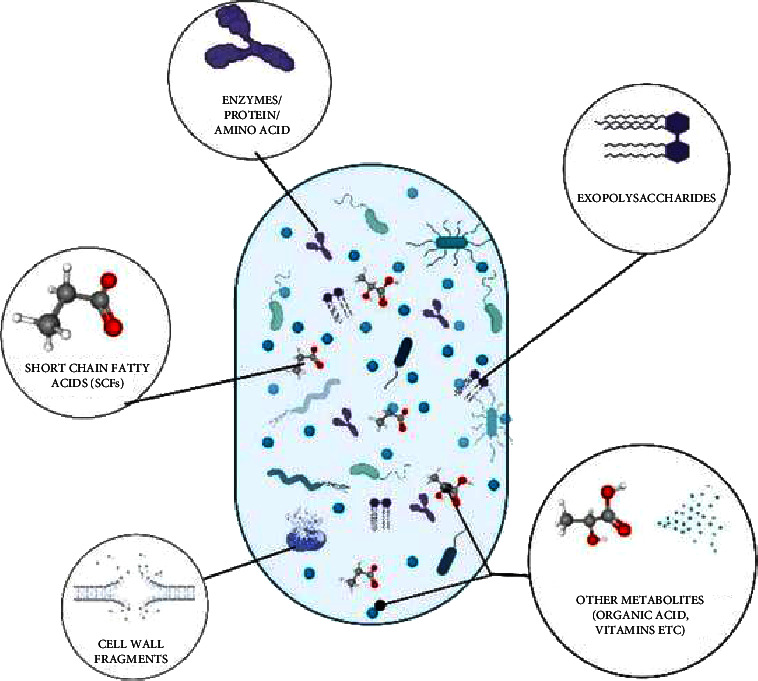
The different types of postbiotics [19].

Based on this, postbiotics are classified as the metabolites generated by the microbiota, such as SCFAs, exopolysaccharides, organic acids, peptides/proteins, and bacteriocins as shown in Figure 1.

#### 2.3.1. Short-Chain Fatty Acids

The main products of intestinal bacterial fermentation are short-chain fatty acids (SCFAs), which have less than six carbon atoms [38]. They are formed when bacteria break down prebiotics and human enzymes ferment dietary fibres to produce energy. SCFA is a suggested type of postbiotics. The most prevalent SCFAs, which are negatively charged carbon-based ions, are propionate (C3), acetate (C2), and butyrate (C4). They result from the bacterial fermentation process. The fermentation of prebiotics such as fructooligosaccharides and inulin leads to the production of SCFA propionate, acetate, and butyrate. They are found in the colon and faeces in a molar ratio of about 60 : 20 : 20 [39]. SCFAs are the main metabolites of the microbiota in the large intestine from the anaerobic fermentation of indigestible polysaccharides such as dietary fibre and resistant starch. SCFAs might influence gut-brain communication and brain function directly or indirectly. The absorption of SCFAs is mediated by substrate transporters, such as monocarboxylate transporter and sodium-coupled monocarboxylate transporter, which promote cellular metabolism. SCFAs are formed when bacteria break down prebiotics and human enzymes ferment dietary fibres to produce energy. SCFAs can also be derived from nondigested proteins or peptides as a substrate.

SCFAs can be used for the synthesis of lipids or glucose. Therefore, SCFAs from gut microbes provide extra energy to host cells, such as colonocytes [40]. Various research studies have linked SCFAs to the positive effects of probiotic Lactobacillus strains. Dhaliwal et al. [41] reported that mice supplemented with *L. plantarum* had increased levels of acetate and butyrate and decreased intestinal permeability and brain monoamine oxidases. Probiotic *L. johnsonii* L531 treatment, which promotes SCFAs, was effective in controlling *Salmonella* infection and keeping metabolic balance in pigs [42]. In a LAB screening to lower cholesterol levels, the strain of *L. plantarum* CECT 7529, which had a high production of propionic and butyric acids, was very good at lowering cholesterol levels [43]. In addition, probiotic strains *L. salivarius* FP25 and FP35 and *L. reuteri* NCIMB had an inhibitory effect on the growth of colon cancer cells, which was due to the production of SCFAs.

#### 2.3.2. Bacteriocins

Bacteriocins are antimicrobial peptides or proteins that are generated by various bacteria, including archaebacteria and eubacteria. Bacteriocins have been used in fermented foods for millennia by humans because of their strong antibacterial effects [44]. Bacteriocins are classified based on their size, mechanism of action, and range of inhibition. Bacteriocins prevent the occurrence and progression of infections in the gut, and they also have other beneficial features, such as heat and pH stability [45]. These postbiotics use three main biofilm defences, which are blocking twitching motility; this biofilm ability is controlled by pili, while flagella activity causes swimming and swarming, disrupting quorum sensing (QS); it influences cell interactions, colonization, and QS signal loss and lowers virulence factors (such as pyocyanin, protease, and rhamnolipid); Pyocyanin helps in biofilm formation and infection detection, and rhamnolipid from *Pseudomonas aeruginosa* [46]. *L. acidophilus* ATCC 4356 produced bacteriocins that stopped *B. subtilis* BM19 from sticking to surfaces and making biofilms [35]. Moreover, they show both narrow and broad inhibitory effects on bacterial growth, drawing attention to their possible therapeutic use as next-generation antimicrobials in lowering the risk of an infectious disease caused by drug-resistant pathogens [47].

#### 2.3.3. Exopolysaccharides

Exopolysaccharides (EPSs) are long and branched chains of sugars or sugar-like molecules that have a high molecular weight mostly produced by lactic acid bacteria (LAB). Depending on the types of sugar units they contain, EPS can be divided into two groups: homopolysaccharides, which have only one kind of sugar unit (such as cellulose, levan, curdlan, pullulan, and dextran) and heteropolysaccharides, which have several different kinds of sugar units (such as xanthan, gellan, galactan, and kefiran) [48]. EPS covers most of the bacterial cells and helps them stick to surfaces and protect themselves from harm. EPS from LAB can have different structures and functions. These natural polymers can be useful for medicine and health because they can affect the immune system, fight against cancer and mutations, prevent oxidation and inflammation, lower blood pressure and cholesterol, and stop harmful bacteria and viruses [49]. Some EPS from *Lactobacillus* bacteria found in fermented durian fruit have antimicrobial and antioxidant properties. EPS can also help with fat metabolism by blocking cholesterol uptake [50]. EPS from *Lactococcus lactis* subsp. lactis increased the levels of antioxidant enzymes such as catalase, glutathione peroxidase, and superoxide dismutase and decreased the levels of lipid peroxidation in the blood and liver of mice [51]. EPS from *Lactobacillus reuteri* Mh-001 had effects on the immune system (Khalil et al. 2018). EPS has many positive effects, especially their anti-inflammatory and antioxidant properties, but how they work is not fully clear yet. EPS is also used by the food industry to make products more smooth, stable, and moist.

#### 2.3.4. Peptides/Proteins

Peptides are a type of postbiotics that the microbiota make. Peptides that kill bacteria (AMP) are postbiotics that make holes in the membranes of bacteria or stop the building of the bacterial wall [52]. Peptides that kill bacteria use different ways, such as stopping the making of big molecules and breaking down the membranes of microbes, to get rid of bacterial infections [53]. Two types of peptides kill bacteria: ribosomal and nonribosomal. Ribosomal peptides made by bacteria can break the membranes of microbes and show strong antibacterial effects in the lab [54]. Peptides are common in all bacteria. Some peptides attack the membrane of the cell, while others attack the inside of the cell and the parts that are sensitive to damage. The ways that peptides kill bacteria are by making the membrane of the bacterial cell more acidic, making holes that let the cell spill out, starting deadly things, such as the hydrolases, that hurt the cell wall, and harming the parts inside the bacteria that are fragile. Bacillus subtilis is a kind of bacteria that makes peptides through its life processes [45].

#### 2.3.5. Organic Acids

A type of postbiotics that can be used to fight against bacteria is organic acids. They are produced by bacteria through fermentation and have two forms of lactic acid, L and D, which can prevent the growth of harmful microbes [45]. Organic acids also prevent the growth of spoilage and disease-causing organisms by increasing the acid concentration and lowering the pH. In addition, organic acids can disrupt the enzymes of pathogens and make them use up all their energy to remove extra proton H+, which causes them to die [55]. When *L. plantarum* produces postbiotics, it makes acetic and lactic acids to help its cells grow [56]. This method of preserving food, which uses different organic acids, could be a way of creating new antibacterial agents for the food industry [45].

The factors that affect the chemical composition of postbiotics include the bacterial species type, culture type and conditions, coculturing strategies for postbiotics production, and postbiotics preparation, processing, and analysis. With advances in metabolomics and bioinformatics, the physiochemical features have been widely explored in determining the physiological functions, i.e., immunomodulation, anti-inflammatory, hypocholesterolemic, antiobesogenic, antihypertensive, antiproliferative, and antioxidant effects [15], which is another classification strategy for describing postbiotics. Well-recognized sources of postbiotics are microbes in the genus *Lactobacillus*, *Bifidobacterium*, *Streptococcus, Faecalibacterium,* and *Saccharomyces*.

### 2.4. Postbiotic Properties That Reflect Potential Local and Systemic Positive Effects in the Host

Recent systematic and meta-analysis reports have revealed prebiotics, probiotics, postbiotics, and faecal microbiota transplantation (FMT) as functional treatment strategies to prevent and treat several clinical complications [16, 57]. Many studies have been conducted using *in vitro* (e.g., diverse cell lines) and *in vivo* (e.g., obese and hypertensive rats) models to evaluate the potential bioactivity and/or health effects of various postbiotics, as will be elucidated in this section.

#### 2.4.1. Antimicrobial Effects

The antimicrobial effect of postbiotics is usually tested against Gram-positive and Gram-negative pathogens such as *Escherichia coli*, *Salmonella typhimurium,* and *Listeria monocytogenes* using the agar disk-diffusion method [58], whereby zone of inhibition indicates positive antimicrobial action. Native-derived EPS exert antagonistic activity against bacterial pathogens, namely, *Vibrio parahaemolyticus*, *Salmonella typhimurium*, *Staphylococcus aureus*, and *Bacillus cereus,* by impairing their cell division [59]. Other antimicrobial mechanisms include surface hydrophobicity, coaggregation, autoaggregation, and diverse functional groups [60]. In a recent advancement in the application of postbiotics, Mohammadi et al. [26] showed that the rich antibacterial and antifungal substances can be harnessed in the development of antimicrobial membrane by bacterial nanocellulose (BNC), a more sustainable material that can be explored in the food industry due to the adsorption capacity and open 3D structure. Postbiotics have gained increasing interest as a safe biopreservative agent; however, to satisfy the guidelines of the European Food Safety Authority (EFSA), further studies are required to evaluate the antimicrobial resistance by determining the minimum inhibitory concentration and the molecular characterization of the antimicrobial resistance genes [58].

A study by Hosseini et al. [61] assessed the antibacterial activity of *Lactobacillus casei* postbiotic extract on *Escherichia coli* in commercially sterilised milk. The study found that the postbiotics of *L. casei* suppressed the growth of *E. coli* in milk. In a different study, Serter et al. [62] found that postbiotics of lactic acid bacteria derived from de Man, Rogosa, and Sharpe (MRS) broth formed larger inhibition zones against pathogenic bacteria than those developed in cow's milk. They also found that the postbiotic-treated groups had fewer *Salmonella spp.* than the control and distilled water groups, and both the postbiotics and 2.1% lactic acid had a bacteriostatic effect on *L. monocytogenes* during the storage period. In comparison to the postbiotics, 2.1% lactic acid had higher reduction (1.8 log10 CFU/g) rates against *Salmonella spp.*

#### 2.4.2. Antioxidative Effects

The buildup of reactive oxygen species (ROS) causes oxidative stress in living beings, which damages biological macromolecules such as DNA, RNA, proteins, and lipids and can result in tissue damage, which can contribute to the onset or progression of detrimental diseases such as obesity, cancer, and neurodegenerative disorders [22]. Postbiotics are considered effective natural antioxidants for preventing oxidative stress caused by free radicals due to their ability to scavenge superoxide anions and hydroxyl radicals. [63] Recently, enzyme digestion revealed the possible enhancement in the antioxidative capability of EPS derived from *Cordyceps militaris*. At a concentration of 1 mg/mL, the 2,2-diphenyl-1-picrylhydrazyl (DPPH) radical, 2,2′-azino-bis-(3-ethylbenzothiazoline-6-sulfonic acid) (ABTS) radical, and hydroxyl radical scavenging activities observed were 30.36%, 69.32%, and 43.82%, respectively. Sourdough rich in postbiotics is obtained by solid-state fermentation with lyophilized water kefir grains culture. The antioxidant properties of the gluten-free sourdoughs against DPPH free radicals vary among different fermentation systems-serial fermentation batches, starter cultures used, and fermentation substrate treatments before the inoculation. The high antioxidant potential can be attributed to the water-soluble peptides, flavonoids, and polyphenols within the fermentation medium [13].

The DPPH radical scavenging assay was used by Aydın et al. [64] to evaluate the antioxidant activity of postbiotics and paraprobiotics in lactic acid bacteria isolated from twelve different hand-made fermented sausages. The results showed that the paraprobiotics' effect on free radical scavenging varied between 5.90% and 18.07%, and the postbiotics' effect was between 5.65% and 76.04%. The findings also showed that the antioxidant capacity of the postbiotics and paraprobiotics is strain-dependent, with the postbiotics having higher antioxidant activity than the paraprobiotics.

#### 2.4.3. Immunomodulatory Effects

The immunomodulatory potential is usually determined by the induction of postbiotics for cytokines production, especially in spleen cells. Individuals suffering from immune-related disorders, caused by either an inadequate or hypersensitive immune system, could potentially experience advantages from probiotic supplementation. It is important to highlight that the effectiveness of probiotics in such cases is heavily influenced by the specific strain and dosage of probiotics used, as well as the conditions being investigated [65]. However, probiotics possess antiallergic properties via the skewing of immune responses by the predominant induction of Th1 proinflammatory cytokines, such as IFN-*γ*, which can, in turn, suppress Th2 cytokines, such as IL-4, and their associated allergic responses [60]. Investigating the immunomodulatory properties of 2 isolates (MBL3 and MBL10) towards IFN-*γ* and IL-4 cytokines production by spleen cells of BALB/c and C57BL/6 mice [60] showed that IFN-*γ* induction is higher in cultures of C57BL/6 spleen cells with the isolate MBL3 (a 65-fold increase over the control) compared to MBL10 (8-fold increase). According to Vale and Mayer [66], lysate and spent media of *L. rhamnosus* Lr-32 have differential effects on the transcription of proinflammatory cytokines encoding genes such as IL-1*β* and TNF-*α*, revealing the increased response of the gingival epithelial cells to *Porphyromonas gingivalis*. Commonly studied proinflammatory cytokines or related genes include interleukin-1 beta (encoding cytokine IL1-*β* to recruit specific immune cell subsets and causes direct tissue damage), interleukin-6 (encoding cytokine IL-6, an inflammatory amplifier), chemokine ligand 8 (encoding CXCL8 chemokine, an inflammatory response mediator), toll-like receptor 2 (encoding the TLR2 receptor, an inflammatory response mediator), toll-like receptor 4 (encoding the TLR4 receptor, involved in signal transduction), and tumor necrosis factor alpha (encoding TNF-*α* cytokine, central mediators of the proinflammatory cascade) [16].

#### 2.4.4. Antiobesogenic Effects

Postbiotics have good absorption, metabolism, distribution, and excretion abilities, which could indicate that they have a great potential to signal diverse organs and tissues in the host, provoking a variety of biological reactions. A wide spectrum of postbiotics is synthesized by the microorganisms from fermented food microbiota exhibiting antidiabetic properties. Youn et al. [67] reported an increase in hesperetin, a potential antiobesogenic agent from inactive precursors (whey and polyphenol-rich citrus pomace extract) bioconverted by kefir lactic acid bacteria (CPB) and fed to C57BL/6J mice on high-fat diets for five weeks. Notable are the reduced body weight gain, adipose tissue weight/body weight ratio, hypertriglyceridemia, and adipocyte diameter, along with increased gene expression related to energy expenditure in adipose tissue (*p* < 0.05). A significant correlation exists between obesogenic biomarkers and the abundance of butyrate-producing and obesogenic gut microbiota [67]. Postbiotic cellular components isolation from kefir lactic acid bacteria can rehabilitate high-fat diet (HFD)-induced dysbiosis and obese characteristic gut microbiome by affecting the adipocyte gene expression in C57BL/6 mice fed an HFD and orally administered 42 mg/kg EPS + 20 mg/kg SLP + 0.5% GSF [68].

#### 2.4.5. Other Effects

Postbiotics offer a safer alternative to probiotics, providing similar health benefits without the risks associated with live microorganism administration [66]. Clinical trials and experimental models have demonstrated potential risks of probiotic treatment, including bloating, flatulence, translocation, bacteremia, fungemia, and antibiotic resistance gene transfer, especially in patients with risk factors such as immunosuppression or concurrent antibiotic use.

Furthermore, postbiotics exhibit various positive effects on both local and systemic levels. These include antihypertensive properties, anti-inflammatory activity, antiproliferative effects on cancer cells, regulation of oncogenes and suppressor genes, antiatherosclerotic activity, cholesterol-lowering capabilities, induction of autophagy, hepatoprotective effects, improved endothelial functions, reduced glycemia, and regulation of the gut microbiota [16, 69]. While the precise mechanisms of these effects are not fully understood, the practical applicability and therapeutic potential of postbiotics in treating various diseases are evident.

## 3. Association of Postbiotics and Gut Microbiome

The gut microbiome plays a crucial role in producing and regulating postbiotics. It comprises a vast ecosystem of microorganisms such as bacteria, viruses, and archaea in the human gut. They play a key role in maintaining human health by impacting human physiology, and they contribute by regulating digestion and metabolism, which could supply various nutrients, regulate energy balance, and also help in the development of the immune system against pathogens [70, 71].

**Figure 2:**
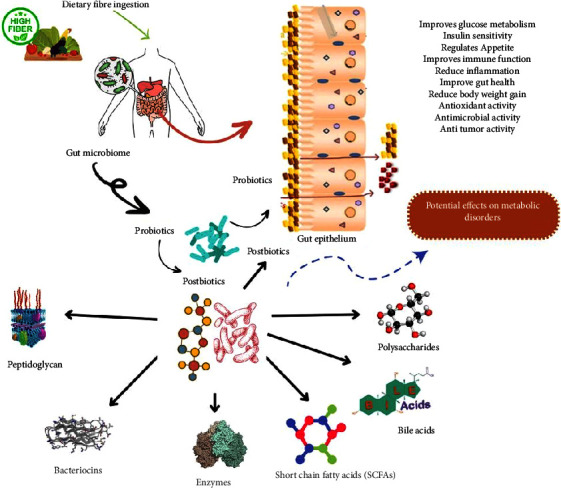
. Association between the gut microbiome, probiotics, and different postbiotics that could have potential effects on metabolic-related diseases (modified from [16]).

Different types of postbiotics exhibit varying effects on the gut microbiome (Table 2), for example, some postbiotics can inhibit harmful bacterial growth while others can stimulate it. Overall, the association between postbiotics and the gut microbiome (Figure 2) is complex, making it difficult to understand. However, understanding the relationship between them is necessary to develop new therapies to improve human health. In addition, the effects of postbiotics on the gut microbiome are determined by various factors such as genetics, diet, different human lifestyles, and the host's environment [16, 75].

Understanding their mechanism of action would help in identifying how these postbiotics would be used effectively for certain health challenges.

### 3.1. Postbiotics in Managing Metabolic Diseases

Metabolic-related diseases such as obesity, dyslipidemia, diabetes mellitus, osteoporosis, and metabolic syndrome are some of the most common metabolic disorders and are a growing global health concern. These diseases are prevalent and they result from metabolic dysfunction [16]. The gut microbiome plays a crucial role in regulating metabolism, and the development of these metabolic-related diseases starts with disturbances in the gut microbiome. By modulating the gut microbiome, postbiotics may play an important role in mitigating the negative effects of metabolic-related diseases [76].

Postbiotics impact metabolic-related diseases by improving insulin sensitivity. Metabolic diseases are characterised by insulin resistance, and postbiotics can improve sensitivity in humans.

Metabolic diseases are also characterised by inflammation, and chronic low-grade inflammation contributes to insulin resistance and metabolic dysfunction [16]. Postbiotics such as SCFAs help in reducing inflammation in the gut and other tissues. SCFAs have anti-inflammatory effects on immune cells (Table 2) [16].

In addition, postbiotics disrupt the production of hormones and neurotransmitters, which may impact energy metabolism. Some postbiotics can increase the production of the glucagon-like peptide 1 (GLP-1), an incretin hormone that promotes insulin secretion and reduces appetite [77]. Other postbiotics may affect the production of neurotransmitters such as serotonin and dopamine [4], which regulate mood and appetite. Table 2 shows the varying effects of different postbiotics on metabolic-related diseases.

#### 3.1.1. Short-Chain Fatty Acids (SCFAs)

SCFAs are produced mainly by fermentation in the gastrointestinal tract. SCFAs have been shown to have numerous beneficial effects on metabolic-related diseases. SCFAs help in increasing GLP-1 levels, which improves insulin sensitivity and reduces body fat [77]. Acetate, an example of SCFA, regulates appetite in the central nervous system [78]. Propionate is another example of SCFA that has shown effects on metabolic-related diseases. This SCFA inhibits the cholesterol synthesis pathway, which helps regulate cholesterol levels, reducing the risk of cardiovascular disease [14]. Propionate has also been shown to have anti-inflammatory activity [79].

#### 3.1.2. Bile Acids

Bile acids are produced in the liver and released into the small intestine to help absorb and digest fat. Metabolic-related diseases have been linked to changes in bile acid metabolism. Bile acids function as signalling molecules that control the metabolism of glucose, lipids, and energy [80]. In metabolic-related diseases, postbiotics that boost the synthesis of secondary bile acids have been demonstrated to enhance insulin sensitivity and reduce inflammation [80].

The modulation of bile acid metabolism by specific postbiotics offers a promising avenue for the prevention and treatment of bile acid-related diseases. By leveraging the gut microbiota's metabolic capabilities, postbiotics emerge as a novel, multifaceted approach to gut and liver health, warranting further investigation and clinical application.

SCFAs, notably acetate, propionate, and butyrate, are postbiotics produced through the fermentation of dietary fibres by gut microbiota. These metabolites have been shown to influence bile acid metabolism significantly. Butyrate, for example, modulates the expression of bile acid transporters in the gut and liver, affecting bile acid enterohepatic circulation and signalling pathways [81]. SCFAs also modulate the farnesoid X receptor (FXR), a nuclear receptor involved in bile acid synthesis regulation, suggesting a therapeutic potential in conditions such as cholestasis where bile acid homeostasis is disrupted [82].

Bacterial enzymes such as bile salt hydrolase (BSH) contribute to bile acid deconjugation, altering bile acid composition and solubility. This enzymatic activity affects bile acid reabsorption and detoxification processes, potentially reducing the risk of gallstones and certain forms of colon cancer [83].

#### 3.1.3. Bacteriocins

Studies suggested that bacteriocins play a role in regulating the gut microbiome and treating metabolic-related diseases. Plantaricin EF (PlnEF) is a type of bacteriocin produced by *Lactococcus plantarum* that has been shown to have anti-inflammatory effects and reduce weight gain in obese humans and mice. It can also help prevent the development of type 2 diabetes and obesity by protecting the gut barrier [84]

#### 3.1.4. Enzymes

Recently, enzymes have been used in various applications because of their substantial low costs for therapeutic strategies. Some enzymes produced by gut microbiota have proven to influence metabolic health. Enzymes produced by LAB help in reducing oxidative stress and inflammation, which are characterised by many metabolic-related diseases. These enzymes include peroxide dismutase, catalase, glutathione peroxidase, and NADH oxidase. Studies have shown that certain strains of *bifidobacteria* and *lactobacilli* can produce these enzymes and help mitigate the effects of metabolic diseases such as lipid peroxidation and inflammatory bowel disease (IBD) [16].

#### 3.1.5. Polysaccharides

Polysaccharides derived from LAB possess various biofunctional abilities such as scavenging a wide range of free radicals to show an antioxidative activity, regulating the gut microbiome, modulating the immune system, and lowering cholesterol levels by binding to free cholesterol [85]. Polysaccharides such as exopolysaccharides (EPSs) have an effect on type 2 diabetes and dyslipidemia [86]. A study by the authors in [87] shows that obesity and metabolic disorders can be prevented and treated with EPS. The study suggests that EPS from *Lactobacillus plantarum* can improve adipocyte glucose absorption through the AS160-mediated pathway, which can be utilized to treat insulin resistance and type 2 diabetes.

#### 3.1.6. Peptidoglycans

These are structural components of bacterial cell walls. As immunomodulatory agents, peptidoglycans have shown promise in mitigating metabolic-related diseases. Studies show that peptidoglycans exhibit anti-inflammatory, antiproliferative, and antitumour activities [16]. However, research on the effect of peptidoglycans on metabolic syndrome-associated dysfunctions such as diabetes, obesity, and insulin resistance is limited.

Postbiotics can all have beneficial effects on metabolic health. Understanding how these postbiotics work is crucial in maintaining good health and can eventually lead to new treatments for metabolic-related diseases.  Table 3 summarises the effect of these postbiotics on different metabolic-related diseases.

## 4. Postbiotics and Food Safety Dynamics

In the host, postbiotics are metabolic byproducts produced by probiotic microorganisms, such as LAB [19]. They are also known as biogenics, metabiotics, or cell-free supernatants [3]. To be considered postbiotics, the products must contain inactivated microorganisms or cell components that benefit host health [17]. Fermentation is a common method for producing postbiotics, for example, pretreating cereal with LAB can increase vitamin B content which is lost during milling or thermal processing [88]. Postbiotics have been shown to have anti-inflammatory, antioxidative, antiobesogenic, antihypertensive, and hypocholesterolemic activities. However, live probiotics can be problematic, especially for the elderly, infants, pregnant women, and people with weakened immune system. Postbiotics are associated with organic acids, vitamins, bacteriocins, hydrogen peroxide, proteins, and peptides. They have been used in the food industry to prevent food spoilage and increase shelf life [45].

Bacteriocins have desirable properties that make them useful in packaging technology to ensure food safety. Bacteriocins are considered Generally Recognized as Safe (GRAS) as they do not affect the gut microbiome and have effective antimicrobial and antibiofilm properties. Examples of bacteriocins include pediocins and enterocins, which have inhibitory effects against *Listeria monocytogenes.*

Ensuring the protection and guaranteeing the safety and quality of food and feed products are crucial for maintaining the overall health and well-being of society. This is especially significant given the increasing demand for these commodities due to the rapid population growth in modern nations. Multiple academic investigations have provided evidence of the advantageous impacts of microorganisms present in the gastrointestinal tract [89]. Nevertheless, the comprehensive safety assessment of probiotics, particularly for vulnerable populations such as neonates, the elderly, and individuals with compromised immune systems, is not yet fully understood. The utilization of probiotics may carry potential risks, including systemic infections and gastrointestinal symptoms, which need to be further examined and understood. As a result, the safety and shelf life of food products are improved, making them potential measures against outbreaks of pathogens and microorganisms that cause food spoilage. Considering the advantages and disadvantages of different bacterial strains, the emergence of postbiotics as a groundbreaking concept in the field of functional food components offers a way to mitigate the potential negative effects of probiotics [90]. However, it is important to consider the specific probiotic strains and fermentation techniques used in the production of postbiotics, as well as any potential safety concerns associated with their consumption. Although postbiotics do not contain live microorganisms and therefore pose no theoretical risk of infection, there is a lack of clinical or epidemiological evidence regarding any potential risks related to their use [91]. Numerous *in vitro* and *in vivo* studies have reported the nontoxic effects of postbiotics mainly supernatant and lipoteichoic acid, on various cells, blood parameters, metabolic biomarkers, and gastrointestinal mucosa [89, 91, 92]. Although the exact mechanisms of action of postbiotics are still not fully understood, they have demonstrated the ability to promote host well-being and provide beneficial outcomes through various pathways. The aforementioned studies have provided evidence supporting the safety of using postbiotics at appropriate doses and concentrations.

Postbiotic mixtures containing antimicrobial properties can also be used to ensure food safety. Studies have shown that synergistic activities between organic acids and organic acids with other metabolites can be beneficial in preventing bacterial growth. For example, a study added postbiotics (produced from *Lactobacillus rhamnosus*) to calcium caseinate and whey protein films, which effectively inhibited the growth of *Escherichia coli, Listeria monocytogenes, Staphylococcus aureus*, and *Salmonella typhimurium* [35]. These findings suggest that postbiotics can be applied to packaging materials to inhibit bacterial growth and improve food safety.

### 4.1. Postbiotics in Food Preservation and Biopreservation

Postbiotics have antimicrobial properties that make them valuable for food preservation. They can inhibit the growth of harmful bacteria such as *E. coli, Listeria monocytogenes, Staphylococcus aureus,* and *Salmonella typhimurium,* offering a natural alternative to chemical preservatives [35]. Incorporating postbiotics into food packaging materials effectively inhibits bacterial growth and extends the shelf life of food products [35]. This is achieved by combining various organic acids, bacteriocins, hydrogen peroxide, proteins, and peptides associated with postbiotics. Postbiotics can also be directly incorporated into food products to enhance preservation. For instance, treating cereals with LAB can increase vitamin B content and inhibit the growth of harmful bacteria [88]. Furthermore, postbiotics play a crucial role in preserving fermented food products such as yoghurt, kefir, and sauerkraut by inhibiting spoilage bacteria. Applying postbiotics to packaging materials can also preserve foods. For example, using cell-free supernatant from *Lactiplantibacillus plantarum* as a preservative for soybeans can increase the product's shelf life by up to 2 months and inhibit mold and bacterial growth [93]. A thin layer of postbiotics can be applied to the surface of the packaging, creating a barrier that prevents the growth of harmful microorganisms.

Utilizing postbiotics as a natural alternative to chemical preservatives, a practice known as “biopreservation,” helps avoid potential negative effects on human health. Biopreservation is an innovative method utilized to prolong the shelf life of food and prevent microbial spoilage. It involves the application of specific microorganisms (primary and secondary cultures) and their antimicrobial byproducts (such as organic acids, hydrogen peroxide, and bacteriocins) to achieve this goal [35]. Research has demonstrated that postbiotic supernatant from *Lactobacillus plantarum* can extend the shelf life of soybeans by 2 months [19]. Bacteriocins, such as nisin produced by *Lactococcus lactis* subspecies *Lactis*, have also been approved as food preservatives and can be found in various products like dairy products, canned soup, and mayonnaise [93, 94]. Postbiotics especially those of low-molecular-weight (H_2_O_2_, organic acids, acetoin, acetaldehyde, etc.) and high-molecular-weight (e.g., bacteriocins) substances demonstrate numerous antimicrobial activities, targeting not only pathogenic microorganisms but also spoilage microorganisms [35]. This characteristic holds significant importance in the food industry. Thus, the biopreservation technique can be applied in the meat, fish, dairy, fruit, and vegetable industries [35].

In the meat and fish industry, postbiotics can be sprayed or applied as a coating depending on the food product. In the dairy industry, it was reported that *Listeria monocytogene*s was sensitive to postbiotics when it was added to milk and stored at refrigerator temperatures. In addition, reports have shown that the fungi population can be reduced when postbiotics are applied to cheese. Postbiotics, specifically phenyllactic acid, an organic acid produced by LAB, have antimicrobial properties and are commonly used in the bakery and dairy industries to inhibit bacterial growth [93]. They can also be used as a solution for preserving fresh produce. Their activity can be enhanced by combining different antimicrobial compounds to replace chlorine-based sanitizers. A study showed that a combination of *Lactobacillus brevis* postbiotics and *Leuconostoc mesenteroides* postbiotics with grape seed extract effectively inhibited the growth of aerobic mesophilic bacteria and mold on vegetable leaves without causing any visible changes to the produce [35].

Table 4 details the use of various postbiotics in food preservation across different food types, elucidating their mechanisms and benefits. Lactic acid is used in meats for its pH reduction and antimicrobial properties, which help extend shelf life and enhance safety [95]. Nisin, an antimicrobial peptide, is applied in dairy products to inhibit spoilage organisms, effectively extending their shelf life [97]. Lysozyme is utilized in bakery products for its enzymatic action that prevents mold growth and extends product freshness [96].

#### 4.1.1. Limitations of Postbiotics in Food Preservation

*(1) Stability*. The effectiveness of postbiotics on food can be affected by changes in the food matrix and storage conditions. For example, the properties of postbiotics can change when interacting with carbohydrates or enzymes, depending on the food matrix. This can lead to a reduction in antimicrobial activity, which may result in bacterial growth in food products [35]. Therefore, it is important to ensure that the postbiotics are stable and maintain their antimicrobial activity during food processing and storage.

*(2) Production*. Producing postbiotics on a large scale can be challenging as it requires specialized equipment and expertise. The conditions required for their production and preservation may vary depending on the microorganism used, which can make the process difficult to replicate. In addition, the cost of producing postbiotics on a large scale can be relatively high [19].

*(3) Safety*. While postbiotics have been considered safe for human consumption, more research is needed to fully understand their potential risks and benefits. For example, it is still not completely clear how postbiotics might interact with other microorganisms in the gut microbiome, and whether they might have any long-term negative effects on human health [98].

*(4) Standardization*. There are currently no standardized methods for producing and measuring the activity of postbiotics. This makes it difficult to compare the effectiveness of different postbiotic products, and it can be challenging to determine which postbiotic products are most effective for food preservation [20].

*(5) Cost*. Production of postbiotics is still in the research phase, so the cost of producing them on a large scale is relatively high.

*(6) Hurdle Technology*. The use of postbiotics alone may not be enough to preserve food products, so hurdle technology, which combines multiple preservation methods, is recommended to ensure food safety. This can include combining postbiotics with other preservation methods such as temperature control, pH control, and modified atmosphere packaging [99].

#### 4.1.2. Benefits of Postbiotics in Food Preservation

*(1) Alternative to Chemical Preservatives*. Postbiotics are a natural alternative to chemical preservatives, which can have negative effects on human health. They do not affect the gut microbiome and have effective antimicrobial and antibiofilm properties.

*(2) Preservation of Food Products*. Postbiotics can be used to extend the shelf life of food products by inhibiting the growth of harmful bacteria [100]. This is particularly useful in the meat, fish, dairy, fruit, and vegetable industries.

*(3) Preservation of Fresh Produce*. Postbiotics can preserve fresh produce by inhibiting the growth of harmful bacteria and fungi. Their activity can be enhanced by combining the antimicrobial compounds that can replace chlorine-based sanitizers.

*(4) Preservation of Fermented Food Products*. They can also preserve fermented food products such as yoghurt, kefir, and sauerkraut by inhibiting the growth of spoilage bacteria. [101].

*(5) Preservation of Food Packaging*. Postbiotics can be applied to packaging materials to help preserve food products. This can include the use of cell-free supernatant from *Lactiplantibacillus plantarum* as a preservative for soybeans, which can increase the product's shelf life by up to 2 months and inhibit mold and bacterial growth [102].

*(6) Addition of Nutrients*. In addition to preserving food, postbiotics can also help to increase the nutritional value of food products by adding vitamins, minerals, and other beneficial compounds.

### 4.2. Postbiotics as an Antibiofilm Agent

Each individual postbiotic possesses a variety of established and emerging food safety functions. These include roles in food biopreservation and packaging, as well as the control and elimination of biofilms formed by foodborne pathogens. In addition, postbiotics contribute to the biodegradation of harmful chemical contaminants, such as mycotoxins, pesticides, and biogenic amines (BAs) [35]. Postbiotics have recently garnered attention due to their well-defined chemical structure, established dosage parameters, extended shelf life, and the presence of diverse signalling molecules that exhibit potential antibiofilm activities [103]. Kefir-derived *Lentilactobacillus kefiri* LK1 although producing higher postbiotics than normal raw milk k-derived *Enterococcus faecium* EFM2 both at an optimum concentration of 25% exhibit antimicrobial and antibiofilm activities by modulating hydrophobicity, autoaggregation, and exopolysaccharide (EPS) production phenotypes and genotypes of bovine mastitis pathogens [104]. These pathogens include *Staphylococcus aureus*, *Enterococcus faecalis*, *Pseudomonas aeruginosa*, and *Escherichia coli*, suggesting the importance of postbiotics in averting the significant challenge these pathogens pose to the dairy industry. Beyond the food application perspective, strong biofilm formation by *Streptococcus mutans* under anaerobic conditions at 37°C for 24 h can be inhibited by microbiota-derived postbiotic mediators which regulate the expression of gtfC, comA, and comX genes [105]. Thus, microbiota-derived postbiotic metabolites have the potential to serve as novel, appealing, and safe ingredients to prevent the development of dental caries, especially those rich in organic acids, fatty acids, and vitamins. The primary mechanisms through which postbiotics combat biofilms involve inhibiting twitching motility, hydrophobicity, disrupting quorum sensing (QS), autoaggregation, exopolysaccharide production, and reducing virulence factors [103, 104]. However, the application of these compounds in the food matrix faces challenges, as factors such as temperature and pH can hinder the antibiofilm effects of postbiotics. To overcome these limitations, encapsulation techniques or incorporation of postbiotics into packaging films can be employed to mitigate the impact of interfering factors. Hence, advanced studies that focus on the safety of postbiotics as antibiofilm agents for several industrial applications are required.

### 4.3. Postbiotics as an Antifungal Agent

The presence of mold in food, feed, and agricultural products can lead to substantial financial losses by causing mold contamination and producing mycotoxins, which are potentially harmful substances capable of causing chronic illnesses and even fatalities [106]. Antifungal screening is primarily conducted to identify potential cultures by filtering the most effective cultures based on their activity and then comes evaluation, which involves quantifying and characterizing the activity. Some of the commonly explored techniques include the disc diffusion test, well diffusion method, microdilution assay, and the fungicidal activity in coculture [107]. The lactic acid bacteria (*Lacticaseibacillus paracasei* ZX1231) isolated from the traditional Chinese fermented wort of Meigui rice vinegar where fungi coexist produce cell-free supernatant postbiotics that exhibit significant inhibitory activities against *Aspergillus Niger*, *Penicillium citrinum*, *Penicillium polonicum*, *Zygosaccharomyces rouxii*, *Talaromyces rubrifaciens*, and *Candida albicans* [108]. Lactic acid bacteria (*Lactiplantibacillus plantarum* and *Pediococcus pentosaceus*) from dry-cured sausages produce surfactants that contain organic acids, phenolic acids, and volatile organic compounds with antifungal activity against toxigenic fungi [107]. Lyophilized and filtered postbiotics derived from *Levilactobacillus brevis* ATCC using the DPPH and ABTS+ methods have antioxidant efficacy and free radical scavenging potential with antimicrobial activity against *P. expansum* [106]. Mycotoxigenic molds causing food degradation pose a significant challenge to food security. Most postbiotics are soluble compounds released by living microbial cells or released upon cell lysis, offering biological action and specific physiological benefits to the host. Antifungal cell-free supernatant postbiotic was rich in four cyclic dipeptides, with cyclo(Phe-Leu) and cyclo(Anthranily-Pro) being a novel bioactive compound produced by LAB in the family Lactobacillaceae that targets the RAS1-cAMP-PKA pathway to hinder fungi filamentation [108]. This implies that the antifungal activity of postbiotics allows for their applications in food preservation and food packaging.

### 4.4. Postbiotics as Nutraceuticals

Nutraceuticals, also known as nonviable probiotics, ghost biotics, or metabiotics, are nutrients that can be used as medicines to provide beneficial nutrients to the host and protect them from diseases. The components associated with nutraceuticals include carbohydrates, lipids, proteins, minerals, and vitamins [109]. Postbiotics as nutraceuticals have potential therapeutic effects and are nutritional. They are more stable than probiotics, and they have positive effects on food allergies and improve immune tolerance [110]. Studies have shown that postbiotics can modulate the immune system, improve gut barrier function, and reduce inflammation, which can help to protect against various chronic diseases such as obesity, diabetes, and cancer [111]. They have also been found to have potential therapeutic effects on neurological disorders such as autism, anxiety, and depression [112]. Postbiotics have also been studied for their potential use as nutraceuticals, which are dietary supplements that have health-promoting properties.

### 4.5. Postbiotics and Functional Foods

Functional foods, which offer additional health benefits beyond basic nutrition, have gained attention among consumers. Postbiotics, containing bioactive compounds, can serve as functional food ingredients due to their health-promoting properties. These foods not only provide essential nutrients but also contribute to the physical and mental well-being of individuals [85]. Studies have shown that postbiotics can positively affect paediatric and neonatal disorders, particularly related to gut health, immunity, and allergies [113]. The intake of postbiotics has been found to enhance the absorption, metabolism, distribution, and excretion of nutrients, indicating their interaction with various tissues and organs in the body [94].

The use of postbiotics in food production can also extend the shelf life of products. A study has found that postbiotics produced from yoghurt cape gooseberry improved the total phenolic content, increasing the antimicrobial and antioxidant properties [114].

When adding antimicrobial agents to foods, sensory properties such as taste, texture, and appearance must be considered. For example, a study by Szydłowska and Sionek [113] showed that adding LAB to cream and semihard cheese did not significantly alter cheese's sensory properties but did affect the sensory properties of sour cream. The study also found that the colour of functional foods may be affected by the addition of postbiotics. For example, when cape gooseberry and postbiotics produced by *E. coli* were added, the colour of the product was not consistent with the control sample. Postbiotics are considered safer than probiotics as they do not contain live microorganisms, but their safety depends on the specific probiotic strain and production technique used. To date, no risks related to postbiotic consumption have been reported [35].

In the development of functional foods, postbiotics play a crucial role by enhancing the health benefits and safety of these products.  Table 5 outlines various postbiotics and their applications in different functional foods. For instance, short-chain fatty acids (SCFAs) are utilized in probiotic yoghurts to enhance gut health and modulate immune responses [115]. Bacteriocins are incorporated into dairy products to inhibit the growth of pathogenic bacteria, thereby extending their shelf life [116]. In addition, exopolysaccharides (EPSs) are used in sauerkraut and kefir for their anti-inflammatory effects and to improve gut barrier functions [117].

#### 4.5.1. Advantages of Postbiotics as Functional Food Ingredients

*(1) Health Benefits*. Postbiotics contain a variety of bioactive compounds that have health-promoting properties, such as anti-inflammatory, antioxidant, and antimicrobial effects.

*(2) Stability*. Postbiotics are more stable than live probiotics, which makes them less sensitive to temperature, light, and pH, making them easy to store and transport [19].

*(3) Safety*. Postbiotics are generally considered safer than live probiotics because they do not involve live microorganisms or show no consumption risks.

*(4) Preservation*. Postbiotics can be used in packaging technology to extend the shelf life of food products by preventing microbial growth.

*(5) Improved Nutrition.* Postbiotics have been shown to enhance the absorption, metabolism, distribution, and excretion of nutrients, indicating that postbiotics interact with various tissues and organs in the body [94].

*(6) Cost-Effective.* Postbiotics can be used as a cost-effective alternative to traditional food preservatives, which can be expensive and may have negative health effects.

*(7) Versatility*. Postbiotics can be used in a wide range of food products, including dairy, meat, fish, fruits, and vegetables [30].

#### 4.5.2. Limitations of Postbiotics as Functional Food Ingredients

The following are a few limitations in using postbiotics as functional foods.

*(1) Stability*. Postbiotics are not as stable as traditional food ingredients, and their bioactive compounds can be affected by factors such as temperature, light, and pH. This can affect their efficacy as functional food ingredients [110].

*(2) Standardization*. There is currently a lack of standardization in the production of postbiotics, which can make it difficult to compare products and determine their effectiveness.

*(3) Safety*. Postbiotics are generally considered safer than live probiotics; however, there is a lack of research on their safety and potential side effects.

*(4) Sensory Properties*. The addition of postbiotics may affect the sensory properties of the food. For example, it may change the taste, texture, and appearance of the food. This may limit their use in certain food products.

*(5) Cost*. Production of postbiotics is a complex process that can be expensive. This can make them less cost-effective than traditional food ingredients [113].

*(6) Limited Research*. The research on postbiotics is still in early stages, and more studies are needed to fully understand their potential benefits and limitations as functional food ingredients.

## 5. Postbiotics as Adjuvants and Their Antiviral Mechanism

Probiotics' role in preventing the common cold and influenza may be premature, as there are numerous strains of probiotics with known immune-modulating effects that are yet to be examined specifically for their impact on these respiratory infections. Clinically investigated probiotics can potentially reduce the incidence and duration of the common cold and influenza, as well as alleviate the severity of symptoms when administered in a dose-dependent manner [118]. Postbiotics, which are the byproducts and metabolites of probiotics, have gained attention as safe alternatives due to reports on the negative clinical and technological effects of probiotics [119]. When consumed in appropriate amounts, postbiotics such as microbial cells, their fractions or metabolites, organic acids, bacteriocins, and enzymes offer a range of health benefits. Although these compounds have consistently demonstrated antioxidant, antibacterial, anticancer, antiallergic, immune-stimulating, anti-inflammatory, and gut microbiota-regulating effects, the antiviral effect through enhancing adaptive and innate immunity remains a growing area of study. Therefore, global concerns arise from emerging viral infections due to their potential to pose a significant threat to public health.

The *in vitro* antiherpes simplex activity of postbiotic lysates or cell-free supernatants produced from *Lactobacillus* strains isolated from Bulgarian fermented milk products influence different stages of viral infection in cell cultures [120]. Postbiotics can impede virus absorption and entry into host cells, as well as inhibit various retroviral reverse transcriptases. The antiviral mechanism of postbiotics encompasses the suppression of viral replication through the initiation of a proinflammatory immune response and the development of Th1-type immunity in infected cells [30]. These responses rely on the production of inflammatory chemokines, cytokines, and interleukins, including TNF-*α*, interferons, IL-23, IL-18, and IL-12, as well as the activation of cytotoxic T-lymphocytes, NK cells, and monocytes/macrophages. The utilization of postbiotics leads to a decrease in the duration of illness, viral shedding, and reoccurrence while postbiotics effectiveness for combating viruses relies on the specific probiotic used for postbiotic extraction and the type of viruses being targeted.

### 5.1. Postbiotics as Adjuvants for COVID-19 Prevention and Treatment

As the COVID-19 pandemic continues, researchers are exploring various ways to prevent and treat the disease. One area of interest is the use of postbiotics as adjuvants for COVID-19 prevention and treatment. Postbiotics have been shown to have antibacterial and antiviral properties and to promote immunomodulatory effects [121]. Studies have shown that postbiotics, along with prebiotics and probiotics, can reduce the progression of the disease and lower the severity of symptoms. They can also block the replication of SARS-CoV-2 by targeting single-stranded RNA [122]. In addition, vitamins C, D, and E, along with zinc and omega-3 fatty acids, have been found to be beneficial for COVID-19 patients [123]. Postbiotics target the epithelial cells of the respiratory tract, the site of SARS-CoV-2 infection. They also promote interaction between gut microbes and the immune system, which is crucial in fighting infections [124]. Products such as kimchi, which contain postbiotics such as bacteriocins, have been found to reduce COVID-19 symptoms [121]. Using postbiotics as adjuvants for COVID-19 has several advantages over live probiotics. Inactivated bacterial cells are administered, reducing the risk for immunocompromised individuals. In addition, postbiotics do not transfer antibiotic-resistance genes and are easier to obtain than live probiotics [85]. Although postbiotics show promise as adjuvants for COVID-19 prevention and treatment, the use of postbiotics as adjuvants is still in the early stages of research, but the findings so far are promising. Further studies are necessary to understand the full potential of postbiotics as adjuvants for COVID-19.

## 6. Future Perspectives of Postbiotics

Postbiotics are considered good alternatives to conventional antimicrobial drugs, with a broad spectrum of activity against different pathogens. In addition to exploring postbiotics, cost-effective and accessible prebiotics can coserve as economical sources of nutrition due to the level of skepticism among individuals regarding the consumption of live bacteria and a greater trust in prebiotics compared to probiotics. Although probiotic-rich foods have greater effectiveness in promoting public health compared to probiotic supplements, without the presence of prebiotics, probiotic bacteria cannot function as intended or as effectively as desired when aiming to deliver health benefits [125]. Also, the potential health benefits of postbiotics as antioxidative agents need to be studied, with a focus on the prevention of oxidative damage in various chronic diseases. These studies are likely to lead to new applications for postbiotics in areas such as functional foods. Postbiotics can also play a significant role in maintaining food safety by inhibiting the growth of foodborne pathogens and improving the shelf life and sensory properties of food products. Developing efficient postbiotic food preservatives and identifying new applications for processing and storage will be a focus of further research in this area. While probiotics have demonstrated health benefits, a few studies have reported side effects and opportunistic infections in humans. However, postbiotics and prebiotics, which possess desirable properties, can potentially offer similar health effects, at a lower risk of microbial invasion, infection, or triggering inflammatory responses and possibly without cytotoxicity [126]. As such, postbiotics and prebiotic products, with their numerous health benefits in disease prevention and treatment, could serve as safe alternatives to live probiotic microbes in functional foods, nutraceuticals, and pharmaceutical products. Overall, the future perspectives of postbiotics in regard to their antimicrobial, antioxidative, and food safety properties are promising. Therefore, further research and development in postbiotics study are expected to lead to important advances in food safety and health with potential applications in fields such as medicine or food science and technology.

## 7. Conclusion

Postbiotics have the potential to be used as functional food ingredients due to their health-promoting properties and ability to extend the shelf life of food products. They have been shown to have anti-inflammatory, antioxidant, and antimicrobial effects, making them beneficial for human health. In addition, they are more stable than live probiotics and considered safer. However, there are also limitations to using postbiotics as functional food ingredients, such as their stability, standardization, safety, sensory properties, and cost. More research is needed to fully understand postbiotics' potential benefits and limitations as functional food ingredients. Overall, postbiotics can be a valuable addition to functional food products, providing health benefits and preservation properties to the food industry.

## Figures and Tables

**Table 1:** Comparative data of each method of postbiotic production.

Method	Advantages	Disadvantages	References
Fermentation	Scalable, enhances bioactivity	Time-consuming, variable yield	[19]
Heat treatment	Quick, effective for sterility	Can degrade sensitive compounds	[18]
Ultrasonication	Efficient cell disruption, fast	May affect the structural integrity of compounds	[17]

**Table 2:** Different types of postbiotics and their effects on the gut microbiome.

Postbiotic	Effect on the gut microbiome	Reference
SCFAs	Protect against intestinal inflammation Maintenance of gut and immune homeostasis.	[71]
Organic acids	Reduce gut PH Inhibit the growth of harmful bacteria	[72].
Bacteriocins	Combat specific intestinal pathogens Improve gut health	[73]
Exopolysaccharides (EPSs)	Improve intestinal barrier function Reduce oxidative stress directly in the gut and thereby positively impact the gut and overall health	[73]
Peptides and proteins	Restore gut microbiota dysbiosis Enhance the proliferation of beneficial gut bacteria Stimulate the immune system Inhibit the growth of harmful bacteria	[74]

**Table 3:** The effect of postbiotics on different metabolic-related diseases.

Postbiotic	Effect on metabolic diseases	Metabolic disease	References
Short-chain fatty acids (SCFAs)	Improves glucose metabolism, reduces inflammation, weight gain, and fat accumulation	Obesity, type 2 diabetes, nonalcoholic fatty liver disease, inflammatory bowel disease, and cancer	[14, 77–79]
Bile acids	Regulates lipid and glucose metabolism and improves insulin sensitivity	Obesity, inflammatory bowel disease, cancer, type 2 diabetes, cardiovascular disease, and insulin resistance	[80]
Bacteriocins	Improves glucose metabolism and insulin sensitivity and reduces inflammation, body weight, and fat accumulation	Obesity and type 2 diabetes	[84]
Enzymes	Antioxidant effect and reduces inflammation	Cancer, inflammatory bowel disease (IBD), and lipid peroxidation	[16]
Peptidoglycan	Reduces inflammation and improves glucose metabolism and insulin sensitivity	Obesity and type 2 diabetes	[16]
Polysaccharides	Improves insulin sensitivity, reduces glucose levels, and improves immune function	Type 2 diabetes, obesity, and insulin resistance	[85–87]

**Table 4:** Application of postbiotics in food preservation.

Postbiotic	Food type	Preservation mechanism	Benefits	References
Lactic acid	Meat	pH reduction and antimicrobial	Extends shelf life and enhances safety	[95]
Nisin	Dairy products	Antimicrobial peptide	Inhibits spoilage organisms and extends shelf life	[97]
Lysozyme	Bakery products	Enzymatic	Prevents mold growth and extends freshness	[96]

**Table 5:** Role of postbiotics in the development of functional foods.

Postbiotic	Functional food	Health benefits	References
Short-chain fatty acids (SCFAs)	Probiotic yoghurts	Enhance gut health and modulate immune response	[115]
Bacteriocins	Dairy products	Inhibit pathogenic bacteria and extend shelf life	[116]
Exopolysaccharides (EPSs)	Sauerkraut and kefir	Improve gut barrier and anti-inflammatory effects	[117]

## Data Availability

The data used to support the findings of this study are available from the corresponding author upon reasonable request.
